# Design and Performance Verification of a Non-Contact Geoelectric Field Sensor Based on a Three-Layer Composite Structure

**DOI:** 10.3390/s26154684

**Published:** 2026-07-23

**Authors:** Shaohong Wang, Da Lei, Qihui Zhen

**Affiliations:** 1 Key Laboratory of Deep Petroleum Intelligent Exploration and Development, Institute of Geology and Geophysics, Chinese Academy of Sciences, Beijing 100029, China; wangshaohong25@mails.ucas.ac.cn (S.W.); zhenqihui@mail.iggcas.ac.cn (Q.Z.); 2 College of Earth and Planetary Sciences, University of Chinese Academy of Sciences, Beijing 100049, China; 3 Innovation Academy for Earth Science, Chinese Academy of Sciences, Beijing 100029, China

**Keywords:** geoelectric field observation, non-contact measurement, high input impedance, low-noise conditioning circuit

## Abstract

Geoelectric field observations play a vital role in geophysical exploration, geological disaster early warning, and underground resource detection. Traditional contact non-polarisable electrodes, which require burial and electrolyte coupling, are hindered by several issues, such as limited adaptability to challenging terrain, significant electrode potential drift, and high susceptibility to environmental interference. Existing non-contact electric field sensors often exhibit insufficient coupling capacitance, poor impedance matching for ultra-weak high-impedance signals, and inadequate low-frequency noise suppression, rendering them unsuitable for the precise acquisition of natural microvolt-level geoelectric field signals. To address these challenges, this study introduces an innovative non-contact geoelectric field sensor with a three-layer composite structure. The sensor operates based on the principle of a parallel-plate capacitor, with a conductive silver paste layer at the top acting as the signal acquisition electrode plate, which forms an equivalent parallel-plate capacitance model with the ground to achieve non-contact capacitive coupling for geoelectric field detection. The intermediate layer uses lead zirconate titanate (PZT) piezoelectric ceramics as a support medium with a high dielectric constant. At the bottom is a silicon-based, flexible, sensitive ground-contacting layer with high elasticity, which allows it to adapt to micro-level surface irregularities, eliminating air gaps between the electrode plate and the ground, increasing plate-to-ground coupling capacitance, and ensuring the stability of the capacitance. The three-layer structure was created using a dry-press sintering integration approach, which eliminates interlayer bonding materials while ensuring consistent dielectric performance and efficient charge transfer. Additionally, a specialised signal-conditioning circuit was designed to match the ultra-high-impedance sensitive unit, utilising the ADA4528-2 ultra-low-noise precision operational amplifier, which achieved low-loss conversion and strong noise suppression for ultra-weak high-impedance charge signals. The circuit simulation results demonstrate that the designed circuit achieves an input impedance of no less than 10 TΩ, an effective operating bandwidth from 0.02 Hz to 20 kHz, and a voltage noise density lower than 1.5 μV/√Hz at 10 Hz, fully covering the ultra-low-frequency effective band of natural geoelectric fields. Field experiments comparing artificial and natural field signals revealed that the proposed sensor could be quickly deployed by simply attaching it to the ground without burial. Its time-domain waveform consistency and frequency-domain component matching were nearly identical to those of commercial standard solid non-polarisable electrodes, with a cross-correlation coefficient greater than 0.98, indicating no significant potential drift or power-frequency interference. By structurally eliminating the inherent electrode potential difference, the sensor offers advantages such as ease of deployment, strong environmental adaptability, high precision for weak signal acquisition, and excellent engineering substitutability. It is well suited for long-term geoelectric field observations in complex field scenarios, including deserts, Gobi areas, and frozen soil regions, and provides a high-performance, novel sensing solution for geoelectric field detection in extreme environments.

## 1. Introduction

The geoelectric field, integral to the Earth’s natural alternating electromagnetic field, provides insights into the spatial and temporal variations in subsurface rock and soil properties, such as conductivity, porosity, and water saturation. This field serves as a crucial geophysical information source for analysing geological structures, exploring oil and mineral resources, tracing groundwater pollution, and monitoring seismic and geological disasters [1,2]. Concurrently, the ongoing development of advanced signal monitoring algorithms in related oil exploration activities such as vibration monitoring for tubing-conveyed perforation [3] demonstrates the broader industry requirement for high-precision data acquisition systems (Shenzhen Jingweiyi Electronics Co., Ltd., Shenzhen, China) in complex subsurface environments. Natural geoelectric field signals are inherently weak, typically registering at the microvolt level, with effective components primarily in the ultra-low- and low-frequency ranges. These signals are prone to interference from environmental factors such as ambient temperature, soil media, and spatial electromagnetic noise, which necessitates that sensing equipment possess high sensitivity, stability, noise suppression capabilities, and adaptability to environmental conditions [4,5]. The accuracy of geoelectric field observation data in terms of authenticity, stability, and continuity is essential for determining the inversion accuracy of underground media, resource exploration precision, and reliability of early warning systems for disasters. High-performance electric field sensors are fundamental to the hardware of geoelectric field observation systems [1].

Currently, contact-type non-polarising electrodes are the most common sensing devices used for outdoor geoelectric field investigations both domestically and globally. The main premise entails creating a conductive circuit by establishing direct contact between the electrode and the soil electrolyte and then measuring the potential difference between the two sites to rebuild the electric field [6]. Non-polarising electrodes have gained popularity over the years because of their simple construction and low cost. However, this technology has inherent limitations that are difficult to overcome in practice. First, there is an inherently poor interface between the internal electrolyte and the metal in the electrode, and this poor contact is extremely sensitive to environmental factors, such as temperature, humidity, and soil salinity. During long-term field observations, the drift amplitude of this contact resistance frequently surpasses the amplitude of natural geoelectric signals, resulting in baseline drift and waveform distortion, which greatly reduces data dependability. Second, to decrease the grounding impedance and maintain successful signal coupling, traditional electrodes must be deeply buried and constantly irrigated with an electrolyte solution. Extreme conditions, such as deserts, the Gobi, dry barelands, and frozen zones on plateaus, cause the electrolyte to evaporate or seep away, resulting in increased grounding resistance, significant signal attenuation, and even observation failure. Third, deep-burial installations are labour-intensive and time-consuming, making quick network deployment, high-density designs, and mobile monitoring problematic. Consequently, the engineering requirements for large-scale, fine-grained geoelectric monitoring are not met. To increase electrode stability, previous studies have sought to reduce contact resistance drift by altering electrode formulations, improving sealing structures, and integrating moisture-retaining media. However, these techniques fail to address the basic difficulties of contact-based measurements, namely, their reliance on medium coupling and limited environmental adaptability [7]. To overcome the limitations of contact electrodes, non-contact capacitive coupling electric field sensing has become a research hotspot in geoelectric observations. Relying on electrostatic capacitive coupling between the plate and ground, this technology enables medium-free, burial-free measurements, eliminating electrolyte dependence while offering convenient deployment, no-contact pollution, and strong terrain adaptability. Current non-contact electric field sensors can be categorised into four types: field mills, optical sensors, MEMS microstructures, and conventional capacitive sensors [8]. Field-milling-type sensors have a quick dynamic response; however, their mechanical construction is prone to wear and has poor low-frequency performance. The use of amplitude demodulation based on rotational frequency limits the performance of ultra-low-frequency (ULF) measurements. As a result, this device is widely used in geomagnetic observation stations and for monitoring of low-frequency (ULF) electrical fields prior to earthquakes [9]. The optical electric field sensor is based on the electro-optic effect and has the advantages of being passively insulated and highly resistant to electromagnetic interference. However, the optical route structure of the system is complicated, and the total cost is considerable. Electro-optic crystals are prone to zero-point and sensitivity drift when the external temperature varies. Long-term unmanned operating scenarios of ultra-low-frequency geoelectric fields at field stations show clear deficiencies in baseline stability and operational dependability. Conventional capacitive sensors have simple structures and controllable costs; however, they generally have low coupling dielectric constants, uncontrollable plate-ground gaps, ultra-high output impedances, and weak signal extraction capabilities. In low-amplitude ultra-low-frequency geoelectric acquisition, effective signals are easily submerged by circuit and environmental noise, resulting in unsatisfactory measurement accuracy for engineering applications [10].

The extraction capability of weak electric field signals is influenced by the coupling capacitance between the sensor and ground surface, as described by the parallel-plate capacitive coupling theory. This capacitance is quantified by the formula:

***C = ε*_0_*ε_r_S/d***

where ε_0_ denotes the permittivity of air, ε_r_ denotes the relative dielectric constant of the medium, ***S*** represents the coupling area of the plate, and ***d*** is the equivalent distance between the plate and the ground surface. Owing to the size and field deployment constraints, expanding the plate area ***S*** to enhance the coupling capacitance is not feasible. Thus, the focus has shifted to optimising high-ε_r_ dielectric materials and reducing the coupling distance ***d*** to improve non-contact sensor performance and address acquisition challenges. Studies have focused on reducing circuit noise, often overlooking the impact of composite dielectric structures and interface fitting characteristics on the coupling efficiency. This oversight complicates the balance between sensor sensitivity and stability, limiting adaptability for high-precision, long-term observations in extreme environments [10,11]. Furthermore, the impedance mismatch between high-impedance capacitive-sensitive units and conventional operational amplifier circuits attenuates ultra-low-frequency signals, posing a challenge to the engineering application of non-contact geoelectric field sensors [10,12].

This study addresses these limitations by developing a three-layer composite non-contact geoelectric field sensor incorporating a silver paste piezoelectric silicon structure. To enhance the dielectric constant of the coupling medium and the sensor–ground coupling capacitance, high-dielectric PZT piezoelectric ceramics (Quanzhou Qijin New Materials Technology Co., Ltd., Quanzhou, China) were used as the medium. The manufacturing process used an integrated dry-pressing and sintering technique, which eliminated the interlayer adhesive media and ensured consistent and stable electrical properties of the resulting device. A silicon-based fitting layer (Dongguan Gupai New Materials Technology Co., Ltd., Dongguan, China) was employed to achieve ground fitting, reduce the coupling distance, and enhance the field-coupling efficiency. Given the ultra-high output impedance characteristic commonly found in electric field sensors, an innovative design of a low-noise and ultra-high input impedance-dedicated signal-conditioning circuit based on ADA4528-2 (Analog Devices, Inc., USA) was developed to solve the problems of attenuation of high-impedance weak signals and noise inundation [13]. The goal is to create a new type of geoelectric field sensing equipment that is not only a non-contact measurement method but also has no polarity difference when compared to solid non-polarised electrodes, is simple to deploy, and has higher sensitivity, stronger anti-interference ability, and coupling capacitance value than existing electric field sensors. This project intends to develop a novel technology solution for high-precision geoelectric field monitoring in exceedingly complicated situations.

## 2. Overall Structure and Working Principle of the Sensor

### 2.1. Overall Structural Design

**Figure 1:**
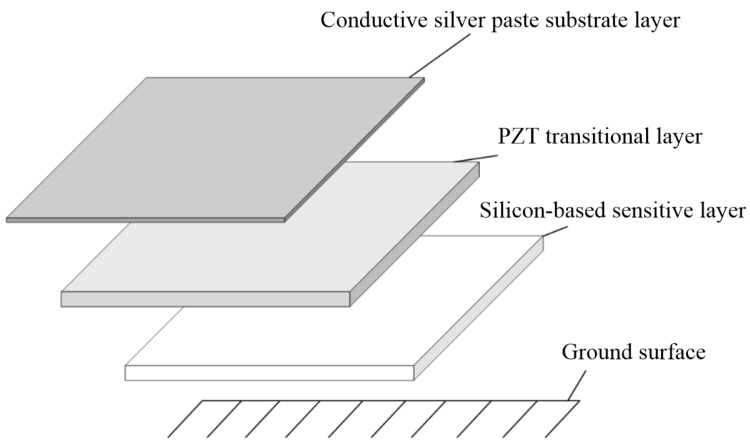
Schematic of the non-contact electric field sensor model.

The proposed non-contact geoelectric field sensor comprises three integral parts: a three-layer composite induction-sensitive unit, a low-noise impedance-matched measurement circuit, and an insulated electromagnetic shielding packaging structure. It features a compact structure, no moving mechanical parts, and no grounding or burial requirements, enabling rapid ground-attached deployment and long-term unattended field observation. The core induction unit adopts a vertically stacked three-layer composite structure consisting of a conductive silver paste base layer (signal acquisition and conductive layer), PZT high-dielectric supporting transition layer, and silicon-based flexible ground-fitting layer from the top to the bottom, as shown in Figure 1. To ensure uniform electric field-coupling and structural mechanical stability, the three functional layers had identical planar projection shapes, with the single-layer thickness precisely controlled at 1 mm ± 0.1 mm to avoid electric field-coupling distortion and signal amplitude deviation caused by structural size errors. Unlike conventional layered assembly sensors, the integrated sintering moulding process eliminates layered assembly defects, achieving superior structural integrity, mechanical stability, and electrical consistency.

For interlayer structure processing, a conductive silver paste layer and PZT high-dielectric supporting layer were fabricated via an integrated dry-pressing and high-temperature silver sintering process. No organic adhesive or transition interlayer exists between the two media, completely eliminating the problems of high dielectric loss, ageing failure, and unstable electrical parameters of adhesive materials, and realising efficient interlayer charge transmission. The bottom silicon-based flexible fitting layer was closely attached to the lower surface of the PZT transition layer. Owing to their high flexibility and ductility, silicon-based materials self-adapt to micro-topographic features, such as tiny ground irregularities and sand gaps, eliminating the coupling loss caused by air gaps, minimising the equivalent sensor–ground coupling distance, and significantly improving the electrostatic coupling capacitance. The sensor was set just above the ground surface at a humidity of 35%. The positive terminal of the digital bridge tester was connected to the sensor output point, whereas the negative terminal was linked to a grounding pin buried in the soil. The sensor-to-ground coupling capacitance was approximately 150 pF. The capacitance value fluctuated with soil moisture and sensor contact, with a range of ±30 pF. It should be noted that variations in the sensor-to-ground coupling capacitance have no effect on the signal output. Because the non-contact electric field sensor is essentially a capacitive sensor, it forms an RC high-pass filter with the input grounding resistance of the measurement circuit, the cutoff frequency of which is affected by the sensor-to-ground coupling capacitance. Proper circuit design ensures that even when the sensor-to-ground coupling capacitance fluctuates within a particular range, the cutoff frequency of the RC high-pass filter created by the measurement circuit remains below the major frequency components of the measured signal. The three-layer induction unit and measurement circuit were entirely packaged in an insulated metal shielding box to suppress the complex spatial electromagnetic interference in the field and improve the purity of weak signal acquisition. The shielding box was completely insulated from the sensor body and ground via insulating supports, achieving electromagnetic shielding without interfering with the accuracy of the geoelectric field measurements.

### 2.2. Core Component Design

#### 2.2.1. Three-Layer Composite Induction Unit

The three-layer composite induction unit constitutes the essential functional element of the sensor, enabling the coupling of geoelectric field energy and the detection of weak signals. Each layer is designed to fulfil specific roles, working in unison to improve the overall efficacy of the sensor in terms of conductive transmission, dielectric coupling, and adaptation to ground conditions. The detailed structural functions and design principles are as follows:

(1) Conductive silver paste-based layers: Serving as the signal output electrode, it is fabricated on the surface of piezoelectric ceramics using screen printing with high-conductivity silver paste materials (Suzhou Xinwei High-Tech Materials Co., Ltd., Suzhou, China). It features excellent conductivity and low contact resistance, which can efficiently collect charge signals generated by coupling and directly connect to the input end of the measurement circuit, thereby reducing signal transmission loss.
(2) PZT Transition Layer: PZT materials, which serve as the central high-dielectric functional medium and mechanical support matrix of the sensor, are characterised by an extremely high relative dielectric constant, excellent electrical stability, and mechanical strength. Compared with traditional sensing media, such as epoxy resins, ceramic sheets, and plastic substrates, PZT materials offer significantly enhanced dielectric performance. This enhancement significantly boosts the ground coupling capacitance of the sensor, even within a limited plate area and structural size, effectively addressing the challenges of low coupling capacity and the extraction of weak signals in conventional non-contact sensors [14]. Furthermore, PZT materials exhibit outstanding temperature stability, with minimal fluctuations in the dielectric parameters across a temperature range of −20 to 60 °C. This stability ensures a consistent electric field-coupling performance under varying operational conditions, thereby enhancing the environmental adaptability and long-term observational stability of the sensor. The integrated sintering process creates a metallurgical bond between this layer and the conductive layer, resulting in an ultra-low interlayer contact resistance and enabling lossless and lag-free charge transmission.
(3) Flexible fitting layers made of silicon-based materials exhibit high elasticity and good insulation. These layers are designed to conform seamlessly to uneven ground surfaces, thereby eliminating air gaps between the plate and the ground. Because air has a relative dielectric constant of only 1, it poses a significant limitation to the enhancement of coupling capacitance. By employing flexible, gap-free fitting, the equivalent coupling distance is significantly reduced, which enhances the overall dielectric coupling performance. Moreover, silicon-based materials are highly resistant to extreme temperatures, ageing, and erosion by wind and sand [5].

These characteristics allow them to withstand harsh environmental conditions, such as those found in deserts, the Gobi region, and areas with frozen soil, preventing structural cracking, detachment, and failure. This ensures that the sensor maintains its structural integrity and performance stability during extended field operations [15].

#### 2.2.2. Low-Noise Measurement Circuit

**Figure 2:**
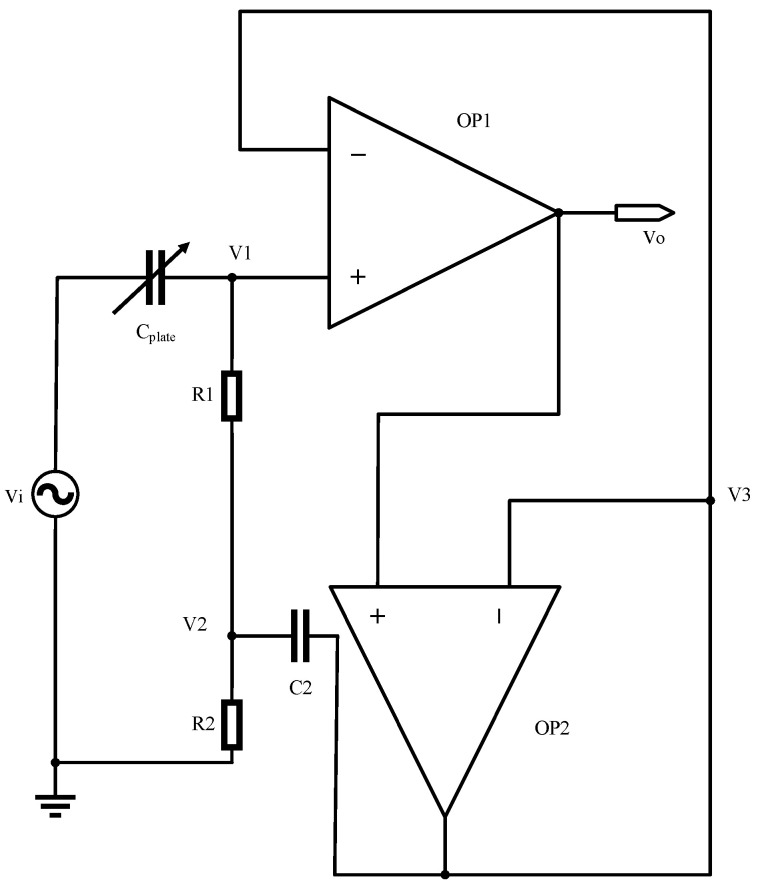
Schematic of the measurement circuit.

Non-contact capacitive electric field sensors are distinguished by their extremely high output impedance and low equivalent capacitance. Ultra-low-frequency weak natural geoelectric signals are vulnerable to attenuation and distortion, owing to impedance mismatch, circuit noise, and limited bandwidth. Conventional operational amplifiers, which typically have low input impedance, high noise density, and inadequate low-frequency performance, are insufficient for the signal-conditioning requirements of these sensors. At a geoelectric signal frequency as low as 0.01 Hz, the output impedance of the sensor can reach 10 GΩ, which far exceeds the input impedance of standard data acquisition cards, leading to significant signal amplitude attenuation and phase distortion when they are directly connected. To address this, the ADA4528-2 high-precision zero-drift and ultra-low-noise operational amplifier from Analogue Devices Inc. (ADI) was selected as the core component for constructing a dedicated signal-conditioning measurement circuit, as shown in Figure 2, and the component parameter table is displayed in Table 1.

This chip offers ultra-high input impedance, ultra-low offset voltage, ultra-low noise density, and stable operation across a wide temperature range, making it ideally suited for the high-precision acquisition of microvolt-level, ultra-low-frequency, and weak geoelectric signals [13].

Following the optimisation of impedance matching, expansion of the low-frequency bandwidth, and adjustment of the noise reduction parameters, the key performance metrics of the proposed measurement circuit were determined. The equivalent input impedance was determined to be at least 10 TΩ, effectively aligning with the ultra-high output impedance of the sensor and resolving the issue of signal attenuation owing to impedance mismatch. The cutoff frequency of the low-pass filter was derived from the major frequency components of the observed signal. In the measurement circuit used in this study, a low-noise fourth-order Butterworth low-pass filter was connected at the Vo point to avoid aliasing of the measured signal with the capacitive signal of high-frequency electromagnetic radiation from the outside. The effective working bandwidth of the circuit ranges from 0.02 Hz to 20 kHz, encompassing all effective signal frequency bands from ultra-low to medium and high frequencies of the natural earth electric field, with no problems of low-frequency signal truncation or high-frequency signal loss [4]. At a typical frequency of 10 Hz, the voltage noise density was measured to be below 1.0 μV/√Hz, which is significantly lower than the effective amplitude of natural geoelectric signals at the 10 μV level. This effectively suppresses the inherent circuit thermal noise, 1/f low-frequency noise, and field electromagnetic noise, thereby preserving weak effective signals. The circuit reliably performs impedance conversion, noise filtering, and voltage amplification of sensor-induced charge signals, achieving a low-loss and high-precision transformation from weak high-impedance charge signals to standardised low-impedance voltage signals. This process provides pure and stable original observation data that are crucial for subsequent advanced data analysis and inversion modelling, such as the accurate prediction of complex petrophysical properties using physically constrained machine learning algorithms [16]. This process provides pure and stable original observation data for subsequent data acquisition, analyses, and inversions.

### 2.3. Working Principle

The proposed sensor facilitates the acquisition of geoelectric signals without contact or drift by utilising an electrostatic capacitive coupling induction mechanism. The operational process was divided into four distinct stages: electric field coupling, charge induction, signal transmission, and conditioning. Upon deployment, the flexible silicon-based layer at the bottom closely conforms to the ground surface. This allows the natural underground geoelectric field to establish a stable electric field distribution on the ground, creating a consistent electrostatic coupling path between the three-layer composite medium of the sensor and ground. The PZT high-dielectric transition layer, known for its strong coupling properties, enables the weak ground electric field to stimulate the composite induction unit, thereby generating an induced charge and converting electric field energy into a charge signal. The top layer, composed of conductive silver paste, serves as a low-loss conductive electrode that efficiently collects the induced charge and transmits it directly to the input of the measurement circuit, thereby minimising signal loss. The ADA4528-2-based measurement circuit then performs ultra-high-impedance matching, suppresses low-frequency noise, and shapes the signal, thereby converting weak high-impedance charge signals into quantifiable low-impedance standard voltage signals. These signals were collected, stored, and post-processed synchronously using a data acquisition card, facilitating continuous and high-precision observation of geoelectric fields. The measurement process does not require electrode polarisation reactions or electrolyte medium participation, and the sensor lacks an inherent contact potential difference, effectively eliminating the issues of potential drift, environmental interference, and medium failure associated with traditional contact measurements, thus achieving high stability and purity in signal acquisition.

## 3. Sensor Fabrication and Circuit Simulation Analysis

### 3.1. Fabrication Process of the Three-Layer Composite Structure

A comprehensive fabrication process was implemented to achieve uniform dielectric properties, structural integrity, and precise interface alignment in a three-layer composite structure, while enhancing the coupling benefits of high-dielectric materials. This process integrates dry-pressing moulding, high-temperature sintering, and screen printing with silver firing. Throughout the procedure, critical parameters such as the raw material ratio, moulding pressure, sintering temperature, and holding time were rigorously controlled to mitigate the performance variations typically introduced by manual assembly. The detailed steps of the fabrication process [17] are as follows:

(1) Preparation and Compression of Raw Materials: An exact quantity of 330 g ± 5 g of the PZT granulated powder was carefully weighed and placed in a designated mould. This material was then compressed into thin sheets, each with a uniform thickness of 1 mm ± 0.1 mm, using a tablet press to maintain substrate flatness.
(2) High-temperature sintering and shaping of materials: The PZT sheets were pressed and subjected to a controlled heating process in a box-type furnace. The temperature was gradually increased to 1100 ± 10 °C at a rate of 5 °C/min. This temperature was maintained for 2 h to ensure the stabilisation of the crystal structure, resulting in a substrate with enhanced dielectric properties of the film.
(3) Surface Polishing Treatment: Once the sheet had naturally cooled, it was subjected to a meticulous polishing process using fine sandpaper. This step was essential for removing burrs, protrusions, and surface imperfections, thereby ensuring smooth and flat interface contact.
(4) Screen printing and silver firing procedures were performed: A uniform layer of conductive silver paste was applied to one side of the PZT sheet using screen printing. Subsequently, the sheets were sintered at 650 ± 5 °C for 15 min. This process facilitates the formation of a metallurgical bond between the conductive layer and PZT substrate, thereby creating an integrated composite structure without adhesives.
(5) Flexible Layer Fitting: The silicon-based sensitive layer was firmly bonded to the reverse side of the PZT substrate, establishing a continuous interface without gaps, thus finalising the construction of the core induction unit.

### 3.2. Measurement Circuit Simulation and Verification

To quantitatively assess the low-frequency response characteristics, gain stability, and noise suppression capability of the designed circuit, and to evaluate its feasibility for acquiring weak geoelectric signals, a comprehensive circuit simulation topology was constructed on the Multisim platform using an official ADA4528-2 chip model. Simulations of the broadband AC gain and low-frequency noise density were conducted to analyse the steady-state electrical performance of the circuit.

**Figure 3:**
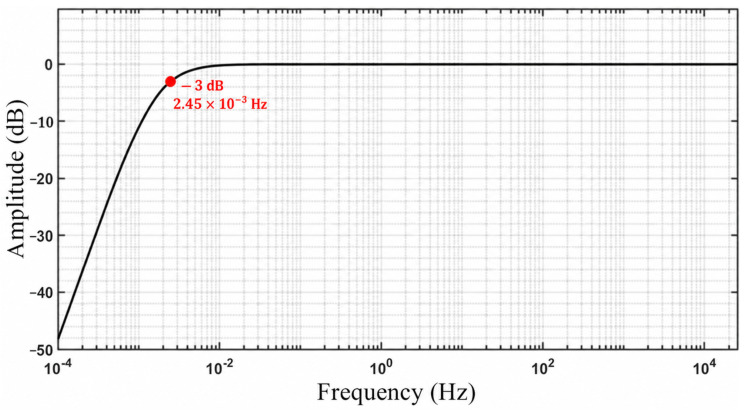
Spectrum simulation of measurement circuit.

Figure 3 shows the simulation results for the AC frequency characteristics of the AC. The circuit achieved a −3 dB cutoff frequency of only 0.0025 Hz, highlighting its superior ultra-low-frequency response. Across the primary geoelectric field operating spectrum of 0.02 Hz–20 kHz, the circuit consistently maintained a voltage gain of approximately 0 dB, exhibiting minimal fluctuations and avoiding significant signal attenuation, amplitude distortion, or phase offset. This design effectively preserves the comprehensive signal characteristics of natural geoelectric fields, thereby resolving the challenges of high low-frequency cutoff and ultra-low-frequency signal loss, which are typically encountered in conventional amplifiers.

**Figure 4:**
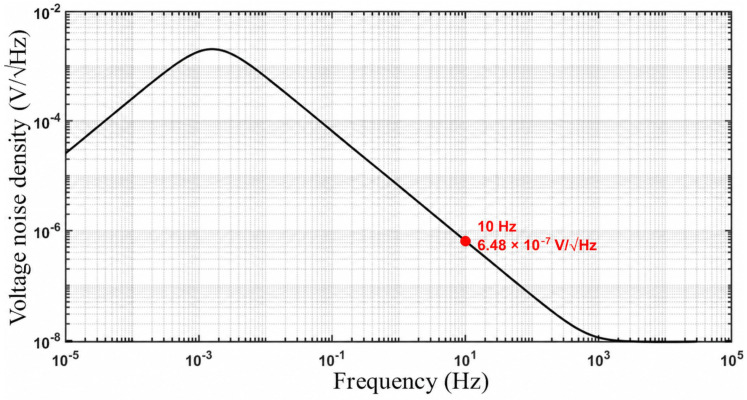
Voltage noise spectrum simulation of measurement circuit.

**Figure 5:**
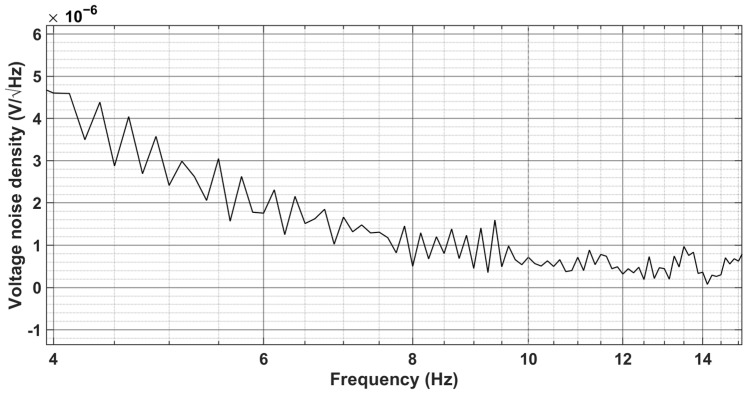
The real test results for measuring the circuit voltage noise spectrum.

Figure 4 and Figure 5 illustrate the simulated and measured results for the low-frequency noise density of the circuit. The low-frequency noise density of the circuit was evaluated using a signal analyser, as explained subsequently. At the characteristic frequency of the 10 Hz electric field, the voltage noise density of the circuit remained steady at 1 μV/√Hz, which is significantly lower than the effective signal amplitude of microvolts of the natural electric field. The intrinsic noise of the circuit does not suppress or interfere with the effective signal. Across the entire operational band, the noise density remained stable, with no abnormal peaks detected, thereby confirming the effectiveness of the circuit’s noise reduction design and its strong anti-interference capabilities for extracting weak geoelectric signals in complex environments. The simulation results comprehensively demonstrate that the designed low-noise measurement circuit offers excellent impedance matching, broad bandwidth coverage, and effective noise suppression, fully satisfying the signal-conditioning requirements of high-impedance non-contact sensors.

## 4. Field Test and Results Analysis

### 4.1. Test Scheme Design

To thoroughly evaluate the measurement precision, signal consistency, and engineering adaptability of the sensor under extreme field conditions, a dual-channel synchronous comparative testing approach was adopted. Standard solid non-polarisable electrodes (Tianjin Bolinger Technology Co., Ltd., Tianjin, China), which have been validated through extensive engineering studies, were employed as reference points. Tests involving the artificial excitation of electric fields have been conducted in an open, interference-free outdoor environment to replicate extreme conditions such as low temperatures, drought, and poor soil quality. The testing location was deliberately chosen to be distant from high-voltage power lines, industrial machinery, and tall structures to prevent interference from external stray electric and magnetic fields. During the tests, the ambient temperature reached as low as −13 °C, and the soil moisture was recorded at 35%, effectively demonstrating the stability of the sensor in terms of temperature and its adaptability to complex environmental conditions.

**Figure 6:**
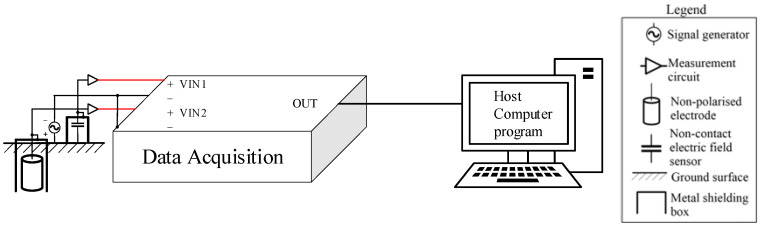
Schematic of on-site measurement system for non-contact electric field sensor.

The configuration of the test system and its wiring topology are shown in Figure 6. The non-contact sensor and standard solid non-polarisable electrode were positioned horizontally in parallel and separated by a distance of 10 m. This setup ensured that both sensing units operated within a consistent artificial electric field, thereby minimising errors owing to variations in the spatial distribution of the electric field. The sensor was completely contained in an aluminium metal shielding box with a thickness of one centimetre. The exterior shell was painted with insulating paint. The shielding box was completely separated from the sensor body, ground, and test circuit. This design effectively reduces interference from spatial electromagnetic radiation and ground loops, thereby enhancing the purity of the acquired signals. The test system comprised a high-precision signal generator, grounding excitation pins, dual-channel synchronous data acquisition card, and isolated battery power supply group. Each device was powered by an independent battery-isolated power supply, which completely eliminated the 50 Hz power-frequency interference and harmonic interference, ensuring the authenticity and reliability of the test data. The initial stage in setting up solid non-polarising electrodes is to excavate electrode pits that are roughly 20–30 cm deep. The electrode body is then positioned vertically at the pit bottom, and fine dirt is filled in and compacted layer by layer to establish a tight connection between the electrode and the soil. Finally, the electrolyte is pumped to completely saturate the soil surrounding the electrode, lowering the contact resistance and ensuring that the electrolytic coupling effect is stable.

During the experimental procedure, a signal generator (Shenzhen Finiris Technology Co., Ltd., Shenzhen, China) was used to produce a consistent sinusoidal alternating current (AC) signal with an amplitude of 2.5 Vpp, designed to replicate natural geoelectric signals across various frequencies. The frequency gradient was systematically varied at 20, 10, 7, 3, 0.7, and 0.3 Hz, thereby encompassing the typical frequency bands (high, medium, low, and ultra-low) of geoelectric fields. Each frequency was maintained for a continuous duration of four minutes to ensure adequate sampling time, thereby enhancing the statistical reliability of the results. The data acquisition card employed high-precision dual-channel synchronous sampling, with the AIN1 channel capturing signals from the proposed non-contact sensor, whereas the AIN2 channel simultaneously recorded signals from the standard solid electrodes. Through the coordination of software and hardware, precise time-axis alignment of the dual-channel data was achieved, eliminating time delays and sampling deviations. The consistency and accuracy of the measurements between the two sensors were assessed and analysed using both the time-domain waveforms and frequency-domain component dimensions.

### 4.2. Time-Domain Signal Comparative Analysis

**Figure 7:**
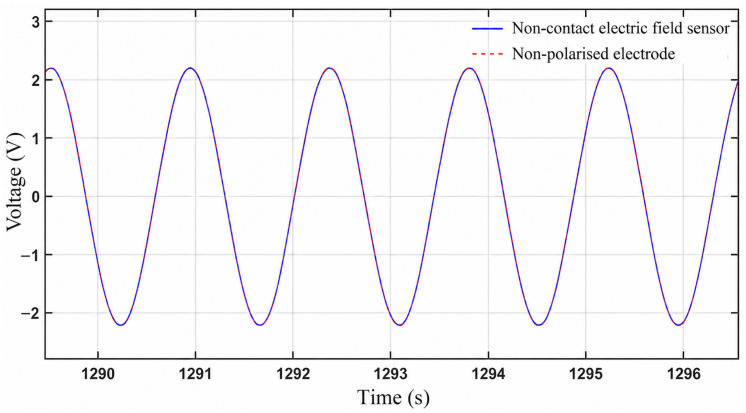
Time-domain comparison of geoelectric signals from non-contact electric field sensor and solid non-polarised electrodes.

The synchronous acquisition results of the time-domain waveforms obtained from the two sensors are presented in Figure 7. The data revealed that the waveforms captured by the proposed non-contact sensor were closely aligned with those obtained from standard solid non-polarisable electrodes at all tested frequencies. These waveforms exhibited regularity and smoothness, were free from distortion, clutter, or power-frequency noise, and maintained a consistent peak-to-peak value of 2.2 Vpp. The slight reduction in amplitude compared to the 2.5 Vpp excitation amplitude of the signal generator was attributed to the voltage division loss caused by the soil impedance, line interface impedance, and grounding resistance. This reduction is a typical engineering error in field measurements that occurs without systematic deviations or regular drifts [5]. The findings confirm that the proposed sensor effectively captures the temporal evolution, amplitude characteristics, and waveform morphology of geoelectric signals, achieving time-domain measurement accuracy and response consistency comparable to engineering-standard electrodes with excellent signal stability.

### 4.3. Frequency-Domain Signal Comparative Analysis

**Figure 8:**
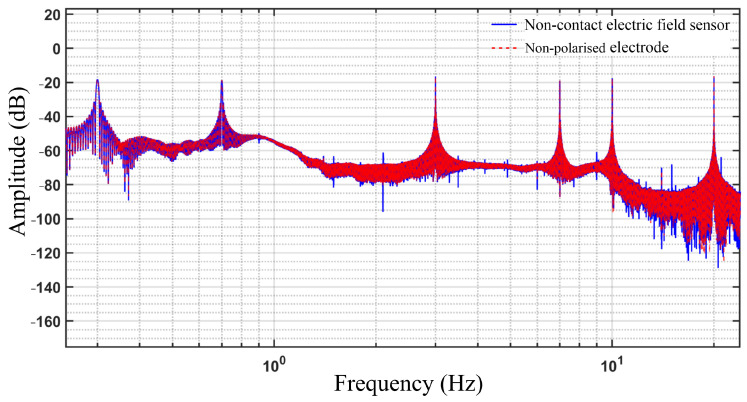
Frequency-domain comparison of geoelectric signals from non-contact electric field sensors and solid non-polarisable electrodes.

To assess the proficiency of the sensor in capturing the signal frequency components, a fast Fourier transform (FFT) was conducted on the original dual-channel time-domain data, which enabled the extraction of the frequency-domain amplitude characteristics. Figure 8 shows the resulting frequency-domain comparisons. The analysis indicated that both signals exhibited pronounced peak responses at all specified characteristic frequency points (20, 10, 7, 3, 0.7, and 0.3 Hz), with no observed peak offsets or frequency loss. The primary and secondary frequency components were perfectly aligned in this figure. The frequency-domain amplitude curves of the two sensors showed a high degree of overall fit, with minimal differences in the main peak amplitudes and only slight variations in the weaker sideband frequency bands. No evidence of stray frequency interference, frequency distortion, or loss of effective components was observed [4]. The test results validated that the proposed non-contact sensor can accurately and comprehensively identify the frequency characteristics of geoelectric signals in various bands. The accuracy of the frequency-domain analysis meets the engineering requirements for geological exploration and electric field monitoring, and the sensor demonstrates excellent broad-spectrum signal acquisition capability.

### 4.4. Natural Field Signal Comparison Analysis

The amplitude of the natural electric field signal is typically in the microvolt to millivolt range. The signal is quickly drowned out by background noise and power-frequency interference. Measuring the natural field signal is essential for assessing the signal detection capabilities of non-contact electric field sensors. Using a three-channel synchronous comparison test scheme, with the non-contact electric field sensor developed in this study as the test object, and simultaneously selecting two commercial solid non-polarisable electrodes (Xi’an Jiaoshan Electronic Technology Co., Ltd., Xi’an, China) from different manufacturers as reference benchmarks, synchronous observation tests of the natural field signal were conducted to verify the non-contact electric field sensor’s capture ability and measurement consistency for weak signals.

The test system was developed and built using the artificial source test system described in Section 4.1, but with an additional solid non-polarisable electrode acquisition channel. The non-contact electric field sensor was installed immediately on the ground without excavation or burial. Two solid non-polarisable electrodes were buried at a depth of 30 cm below the ground surface in accordance with the normal technical criteria, and wet soil was filled around the electrodes to ensure proper coupling. The test location was an open, naked outdoor region free of high-voltage power transmission lines, industrial structures, and mobile phone base stations. During the test, the ambient temperature was 35 °C, and the surface soil moisture was 45%.

**Figure 9:**
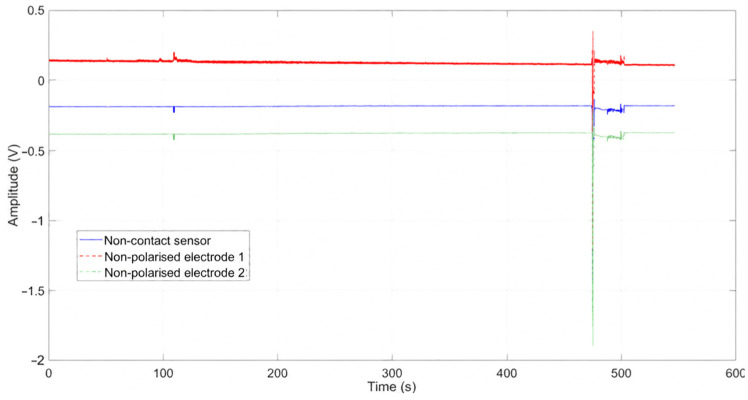
Time-domain comparison of natural electric field signals obtained using three types of sensors.

Figure 9 shows the comparative results of the time-domain waveforms of the natural field signals captured concurrently by the three sensors. The time-domain waveforms of the two solid non-polarisable electrodes and the non-contact electric field sensor were similar in terms of the overall waveform shape. The non-contact electric field sensor can perfectly capture time-domain dynamic changes in the natural geoelectric field, as evidenced by a completely constant trend in the signal fluctuation, fluctuation period, and peak moments. The output signals of the three sensors in the natural field background were generally at the millivolt level based on the signal amplitude, which is compatible with the characteristics of weak signals in the natural geoelectric field. At approximately 475 s, a strong peak formed in the waveforms of all three routes. This was because the staff actively touched the soil at the test location. All three types of sensors detected this transient peak, and the peak occurrence periods were perfectly synchronised, demonstrating that the non-contact electric field sensor can respond to electric field changes as quickly as typical solid non-polarised electrodes.

**Figure 10:**
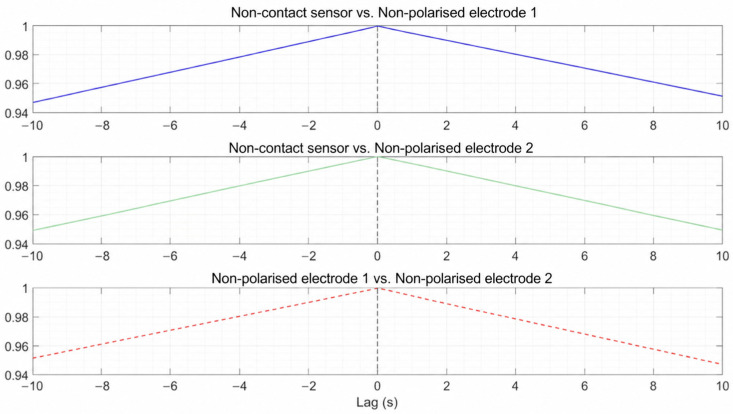
Inter-correlation comparison chart of natural earth electric field signals measured by the three types of sensors.

Figure 10 shows the normalised cross-correlation analysis results of the three-signal synchronous acquisition, which were used to quantitatively evaluate the waveform consistency and time delay characteristics of the measurement results obtained using the non-contact electric field sensor and solid non-polarisable electrodes. The cross-correlation coefficient ranges from −1 to 1. The closer the coefficient is to one, the more similar the waveforms of the two signals. The peak value represents the time delay, which reflects the phase difference between the two signals. The three sets of comparison curves in the figure show that the cross-correlation curves of the non-contact electric field sensor and the two solid non-polarisable electrodes both peak at zero time, and the normalised cross-correlation coefficient at zero time is greater than 0.98. The cross-correlation coefficient between the two solid non-polarisable electrodes was also greater than 0.98, approaching the ideal synchronous state. The foregoing results show that the natural terrestrial electric field signals detected by the non-contact electric field sensor, as well as the measurement results of typical solid non-polarisable electrodes, have a constant waveform shape and an extremely low time-phase delay. The three signals exhibited excellent synchronisation and consistency. Cross-correlation analysis quantitatively confirmed that the non-contact electric field sensor developed in this study can accurately reproduce the time-domain fluctuation characteristics of the natural terrestrial electric field, and its measurement results are comparable to those of commercial standard solid non-polarisable electrodes.

**Figure 11:**
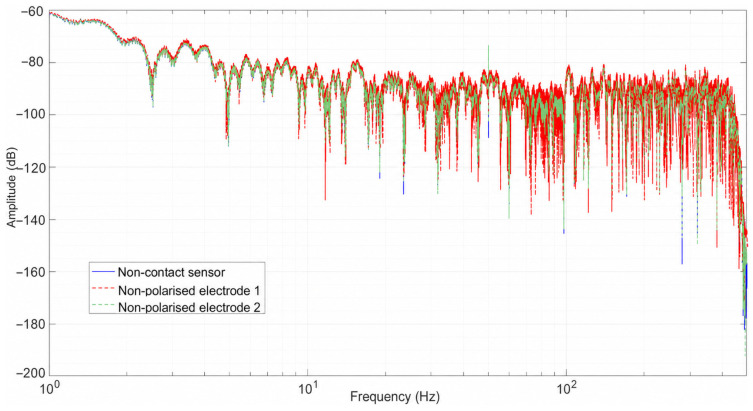
Frequency-domain comparison graph of natural earth electric field signals obtained by three types of sensors.

To investigate the signal response characteristics and noise levels of the three-channel sensors throughout the entire frequency range, the original time-domain data were subjected to a rapid Fourier transform to generate a frequency-domain amplitude spectrum. Figure 11 shows the comparative results in the frequency domain for the two models. The spectral curves of the three-channel sensors were virtually identical based on the overall frequency spectrum distribution, with identical principal energy distributions and distinct peak locations. This suggests that non-contact sensors have better consistency in the transmission properties of natural field components than standard solid, non-polarisable electrodes. At 50 Hz, the three-channel spectra exhibited apparent power-frequency interference peaks with almost identical peak amplitudes, indicating the power-frequency interference intensity in the environment. This also confirms that the sensitivity of the three-channel sensors to the power frequency of the electric field is the same. A more precise evaluation of the signal-to-noise ratio (SNR) was conducted at the 50 Hz power-frequency interference component. The results indicated that the non-contact electric field sensor developed in this research exhibited a 50 Hz power-frequency SNR of 20.3 dB. In comparison, the reference solid non-polarised electrode 1 showed an SNR of 20 dB, and the solid non-polarised electrode 2 had an SNR of 20.22 dB. The SNR values for all three sensing devices were comparable, indicating that the non-contact sensor is equally effective in minimising power-frequency interference and capturing useful signals as conventional commercial solid non-polarised electrodes. Additionally, it performs similarly in detecting weak geoelectric field signals in practical applications.

## 5. Performance Advantages of the Proposed Sensor

Incorporating structural innovation design, circuit simulation characteristics, and field comparative test results, the proposed three-layer composite non-contact geoelectric field sensor exhibited notable technical superiority over traditional contact electrodes and conventional non-contact electric field sensors. This superiority is evident in its structural mechanism, sensing performance, environmental adaptability, and engineering applications, which are discussed in the following sections.

(1) The sensor demonstrates exceptional adaptability to various environmental conditions and is easy to deploy: Unlike traditional electrodes that require burial and electrolyte irrigation, this sensor can be placed directly on the ground to measure soil moisture. This innovation effectively resolves the challenges associated with electrode burial and electrolyte depletion in challenging terrains, such as deserts, the Gobi region, and permafrost areas, thereby significantly reducing the complexity of field operations.
(2) The sensor is characterised by its precise measurement capability and stable signal output: It inherently lacks an electrode potential difference, which effectively prevents signal drift and distortion due to environmental influences, such as temperature fluctuations and soil conditions. The integrated composite structure ensures consistent dielectric performance and uniform electric field coupling, thereby guaranteeing the reliability of long-term measurements.
(3) Superior weak-signal extraction capability: The PZT composite structure, with its high dielectric constant, significantly enhanced the device coupling capacitance. This improvement, when paired with an ultra-high impedance and low-noise measurement circuit, effectively mitigates the challenges of weak signal intensity and noise interference, which are common in traditional non-contact sensors. Consequently, this configuration allows for the precise detection of microvolt-level ultra-low-frequency geoelectric signals.
(4) The model demonstrated a remarkable ability to resist interference from other signal types: Its insulated metal shielding packaging structure effectively blocks the spatial electromagnetic interference encountered in field environments. This, combined with a low-noise circuit design, facilitates the acquisition of high-purity signals, even under complex field conditions.
(5) The materials demonstrate a high degree of engineering substitutability: Tests conducted in both the time and frequency domains have shown that their measurement capabilities are closely aligned with those of traditional solid and non-polarisable electrodes. These materials can directly replace conventional contact measurement systems and are adaptable to various geoelectric field exploration and monitoring applications.

## 6. Conclusions

This study presents an innovative solution to the technical challenges faced by traditional contact geoelectric electrodes, such as limited environmental adaptability, significant potential drift, and complex deployment, as well as the limitations of current non-contact sensors, including low coupling efficiency, poor impedance matching, and inadequate weak-signal extraction capability [14,15]. We developed a novel integrated three-layer composite non-contact geoelectric field sensor characterised by a silver paste–PZT–silicon structure. The use of a high-dielectric PZT functional medium and a flexible, gap-free fitting structure enhances the sensor–ground electric field-coupling capacitance at the mechanistic level, effectively addressing the low coupling capacity issue of traditional non-contact sensing structures. The integrated dry-pressing and sintering moulding process eliminates interlayer bonding defects, ensuring long-term stability in both structural and electrical performance. An ultra-low-noise signal-conditioning circuit based on the ADA4528-2 was designed to complement the high-impedance sensing unit, thereby resolving challenges related to ultra-weak-signal attenuation and noise interference. Circuit simulations confirmed the superior electrical characteristics of the sensor, including ultra-high input impedance, broadband response, and ultra-low noise levels. Field comparative tests conducted under extremely low-temperature and dry conditions demonstrated that the time- and frequency-domain characteristics of the proposed sensor closely matched those of commercial standard electrodes, with the measurement accuracy and stability fully satisfying the engineering requirements.

By removing the electrolyte medium and the buried deployment mode, the sensor effectively addressed the issue of electrode potential drift, thereby overcoming the challenges associated with geoelectric field observation in extreme terrains. This sensor offers easy deployment, strong resistance to interference, high measurement precision, and outstanding long-term stability, making it ideal for extended field observations in complex environments such as deserts, Gobi areas, frozen soil, and arid bare land. A future study will be conducted in three areas: structural optimisation, field verification, and engineering promotion. First, it will concentrate on optimising the structure and materials. The capacitance value of the sensor for ground coupling will be improved further by optimising the preparation process of PZT composite high-dielectric-constant materials in terms of thickness and the dielectric constant. Meanwhile, the flexible piezoelectric sensing technology approach will be used to investigate the fabrication method of ultra-thin PVDF composite film layers with great flexibility and durability. At the same time, active shielding will be implemented to further minimise the voltage noise density of the measuring equipment. Based on this, the engineering sample preparation will be completed, and long-term field tests in typical extreme environments such as deserts and the Gobi will be conducted to systematically verify the sensor’s temperature and humidity durability, as well as anti-interference performance. Finally, the preparation technique will be standardised and pushed for engineering applications, with the goal of eventually replacing solid non-polarised electrodes at existing exploration sites. Subsequent studies can improve the sensor’s coupling sensitivity and low-frequency resolution by optimising the PZT material ratio, improving the structural size parameters, and incorporating microstructure sensitization design. Simultaneously, long-term fixed-point field tests will be performed, the temperature drift correction algorithm will be improved, and the standardisation, engineering development, and large-scale application of this type of non-contact high-performance geoelectric field sensor will be promoted, resulting in new high-precision and highly adaptable sensing technology support for geophysical exploration, geological disaster warning, and resource and environment detection.

## Figures and Tables

**Table 1:** Component parameters of the measurement circuit.

Parameter	Symbol	Value	Unit
Plate coupling capacitance	C_plate_	150	pF
Feedback resistor 1	R_1_	10	GΩ
Feedback resistor 2	R_2_	10	GΩ
Feedback capacitance	C_2_	100	μF

## Data Availability

The original contributions presented in this study are included in the article. Further inquiries can be directed to the corresponding author.
